# A Combined Physical Activity and Multi-Micronutrient Supplementation Intervention in South African Primary Schools: Effects on Physical Activity, Fitness, and Cardiovascular Disease Risk Factors

**DOI:** 10.3390/children12101352

**Published:** 2025-10-09

**Authors:** Siphesihle Nqweniso, Cheryl Walter, Rosa du Randt, Larissa Adams, Johanna Beckmann, Danielle Dolley, Nandi Joubert, Kurt Z. Long, Ivan Müller, Uwe Pühse, Harald Seelig, Peter Steinmann, Jürg Utzinger, Christin Lang, Markus Gerber

**Affiliations:** 1 Department of Human Movement Science, Nelson Mandela University, Gqeberha 6031, South Africa; cheryl.walter@mandela.ac.za (C.W.); rosa.durandt@mandela.ac.za (R.d.R.); larissa.adams@mandela.ac.za (L.A.); danielle.dolley@mandela.ac.za (D.D.); nandi.joubert@swisstph.ch (N.J.); 2 Department of Sport, Exercise and Health, University of Basel, CH-4052 Basel, Switzerland; johanna.beckmann@unibas.ch (J.B.); ivan.mueller@unibas.ch (I.M.); uwe.puehse@unibas.ch (U.P.); harald.seelig@unibas.ch (H.S.); christin.lang@unibas.ch (C.L.); markus.gerber@unibas.ch (M.G.); 3 Swiss Tropical and Public Health Institute, CH-4123 Allschwil, Switzerland; kurt.long@swisstph.ch (K.Z.L.); peter.steinmann@swisstph.ch (P.S.); juerg.utzinger@swisstph.ch (J.U.); 4 University of Basel, CH-4001 Basel, Switzerland

**Keywords:** cardiovascular health, COVID-19, micronutrients, physical activity, randomized controlled trial, school-based intervention, schoolchildren

## Abstract

**Highlights:**

**What are the main findings?**

- School-based physical activity (PA) interventions, alone or combined with multi-micronutrient supplementation (MMNS), did not increase daily PA and showed mixed effects on cardiometabolic health in South African children aged 6–12 years.
- The MMNS improved cardiorespiratory fitness (CRF) and reduced blood pressure but was linked to adverse metabolic changes (triglycerides and high-density lipo-protein).

**What are the implications of the main finding?**

- Nutritional supplementation may hold potential for improving CRF and blood pressure in school-age children, though possible metabolic trade-offs require careful consideration.
- PA interventions alone may be insufficient to change activity patterns in marginalised settings, highlighting the role of broader environmental and contextual barriers.

**Abstract:**

**Background/Objectives**: Declining physical activity (PA) and cardiorespiratory fitness (CRF) in children are global public health concerns, particularly in populations experiencing urbanization and economic transition. This study investigated the effects of a school-based intervention on PA, CRF, and cardiovascular disease (CVD) risk factors in children aged 6–12 years from marginalized communities in Gqeberha, South Africa. **Methods**: A cluster randomized controlled trial was conducted in four schools, with participants randomly assigned to one of the following four arms: (i) PA and multi-micronutrient supplementation (MMNS); (ii) PA and placebo; (iii) MMNS; or (iv) placebo (control). A total of 1151 children were assessed at baseline (T1), 1003 at post-intervention (T2), and 549 at follow-up (T3). PA was measured using accelerometers. Secondary outcomes included CRF (20 m shuttle-run) and CVD risk factors (i.e., anthropometry, blood pressure, glycated hemoglobin [HbA1c], and lipid profile). Mixed linear models adjusted for baseline characteristics were used. **Results**: None of the interventions significantly improved daily PA. From T1 to T2, the MMNS arm significantly increased CRF, while PA + MMNS reduced HbA1c. However, MMNS alone increased triglycerides, and PA + placebo increased low-density lipoprotein (LDL). From post-intervention (T2) to follow-up (T3), the MMNS arms significantly reduced blood pressure. Yet, the PA + MMNS arm increased body fat percentage and decreased high-density lipoprotein (HDL). **Conclusions**: While MMNS showed promise for improving fitness and blood pressure and PA + MMNS reduced HbA1c, adverse metabolic changes emerged. The results should be interpreted with caution due to the short intervention span and COVID-19 disruptions during the second year of the intervention.

## 1. Introduction

Physical activity (PA) and cardiorespiratory fitness (CRF) levels are declining rapidly among children and adolescents [1], with significant implications for both short- and long-term health [2]. In parallel, cardiovascular diseases (CVDs) are on the rise, particularly in low- and middle-income countries (LMICs) undergoing rapid urbanization and economic transition [3]. Since PA is an independent risk factor for CVD [4], and early-life risk factors often track into adulthood [5], these trends indicate the emergence of a public health crisis in LMICs [2,6].

Childhood PA is a critical determinant of both childhood fitness and adult cardiovascular health [7]. Schools represent an optimal and often cost-effective setting for promoting PA as a component of a healthy lifestyle [8,9]. Compulsory physical education in schools ensures that all children, including those who are typically less active, overweight, or unfit and may not engage in voluntary sports, have opportunities for physical engagement. In South Africa, despite physical education being part of the school curriculum, its implementation is often inadequate, particularly in historically marginalized communities [10,11]. In these settings, physical education is often not prioritized compared to academic subjects and faces significant challenges such as insufficient funding, limited physical resources and equipment, and a scarcity of qualified physical education teachers for the large class sizes [12]. The low quality of physical education implementation in low-resourced schools places children at a higher risk of suffering the consequences associated with low PA, including overweight/obesity, low physical fitness, and compromised cardiovascular health extending into adulthood [7,13,14].

Despite improvements in PA levels reported in the 2022 Healthy Active Kids South Africa (HAKSA) report card, from 48–52% in 2018 to 60–73% children meeting PA recommendations, these figures may be overly optimistic due to the lack of nationally representative data [15]. The COVID-19 pandemic further constrained PA opportunities due to the strict lockdown measures implemented in South Africa, which adversely affected children’s opportunities to meet PA recommendations [16]. The World Health Organization (WHO) noted that the pandemic exacerbated inequalities in access and opportunities for being physically active, especially in resource-constrained communities with poor infrastructure and unsafe public spaces [17]. Nevertheless, the importance of interventions promoting PA, especially in marginalized communities has been emphasized [15], with research supporting the potential of school-based interventions to improve PA and fitness in youth [1,8,18].

While previous school-based health interventions have focused on fitness [19], body composition [20], and CVD risk profiles [21,22], most were conducted in high-income countries. South African studies have incorporated combinations of PA and nutrition [23,24,25], and some have included PA, nutrition, and hygiene [26]. The role of micronutrients in enhancing physical performance was demonstrated in a study including adult men [27], and benefits have also been observed in school-age children. For instance, in South Africa, 5–11-year-old children with mild iron and zinc deficiency from low-socioeconomic areas receiving a micronutrient powder added to a daily portion of maize porridge increased their weight-for-age, serum ferritin, and iron levels compared to the control group [28]. Healthy 7–10-year-old children from middle-socioeconomic areas in India improved aerobic capacity and endurance after receiving fortified choco-malt beverage powder [29]. In Brazil, a reduction in total cholesterol, LDL cholesterol, and glucose was reported in children aged 9–13 years after taking 2–3 micronutrient bars (Nestrovit™) for 6 weeks, suggesting micronutrients play a role in reducing CVD risk factors [30].

Yet, few studies have investigated the combined effects of PA and multi-micronutrient supplementation (MMNS) in South African schoolchildren. Multi-component health interventions are increasingly recognized as more effective in LMICs due to the dual burden of disease [31].

Owing to the paucity of evidence but considerable potential of school-based health interventions, this paper addressed this evidence gap by conducting a cluster-randomized trial which included a PA and MMNS interventions in primary schoolchildren from marginalized communities in Gqeberha, South Africa. The first aim was to assess the effectiveness of these interventions (alone or in combination) on schoolchildren’s daily moderate-to-vigorous physical activity (MVPA) levels. The second aim was to assess the effect of the interventions on CRF (VO_2_max) and CVD risk factors (blood pressure, low-density lipoprotein cholesterol (LDL-C), high-density lipoprotein cholesterol (HDL-C), etc.).

## 2. Materials and Methods

### 2.1. Study Design

The study was designed as a double-blind, cluster-randomized controlled trial that took place in four schools from marginalized peri-urban communities in Gqeberha, Eastern Cape, South Africa. The study forms part of a larger study (KaziAfya) that aimed to examine the effects of a school-based health intervention (PA, MMNS, or both interventions combined) on growth, health, and well-being in schoolchildren from three African countries; South Africa, Tanzania, and Côte d’Ivoire [32]. The study was registered with the International Standard Randomized Controlled Trial Number (ISRCTN), registry (ISRCTN29534081). The current study is based on data collected from South Africa, with data assessed at three time-points; baseline T1 (February–April 2019), T2 (September–November 2019), and T3 (August–November 2021).

### 2.2. Participants and Sampling

Children aged 6–12 years from four primary schools (41 classes) were invited to participate in the study. All schools were selected from quintile 3 schools in Gqeberha, with quintile 1 schools representing the “poorest” quintile and quintile 5 schools representing the “least poor”. Quintile 3 schools are the lowest ranking quintile schools in the area. Children were eligible for inclusion if they were between the ages of 6 and 12 years, in grades 1 to 4, provided written informed consent from parents/guardians, not participating in any other clinical trial or nutrition, and not suffering from any clinical condition preventing participation in physical education or absorption of the supplement, as determined by medical personnel. For sample size calculation, a total sample of 1096 children were needed as indicated by power calculations (calculations based on G*power 3.1: alpha error probability = 0.05, power = 0.80, number of groups = 12, number of measurements = 3) [32]. Based on a priori power analysis, in each country, the final target sample was increased to 1320 children, assuming a yearly dropout rate of 10%, with 330 children allocated to each of the intervention arms.

Four schools were randomly selected, and following baseline assessment, intervention and control classes were randomly allocated to one of four intervention arms using a computer-generated randomization code. The intervention arms were the following: (i) PA and MMNS; (ii) PA and placebo; (iii) MMNS only; and (iv) placebo, which served as the control group (Table 1).

### 2.3. Randomization to Intervention Arms

To ensure double-blinding, intervention arm randomization was conducted by the project coordinator (C.L.), who was not otherwise involved in recruitment or data collection, while the local coordinator (S.N.) remained blind to MMNS group allocation. As depicted in Table 1, children were randomly allocated by class to one of the four intervention arms via stratification to ensure equal representation at each school across the grade levels.

Children in the PA arms received the *KaziKidz* toolkit, which aligns with South Africa’s Curriculum Assessment Policy Statements (CAPS) [33] (see https://www.kazibantu.org/kazikidz/, accessed on 22 July 2025). This teaching and learning toolkit was developed in accordance with the criteria and guidelines of two key UNESCO programs, namely, the Quality Physical Education (QPE) (https://www.unesco.org/en/quality-physical-education, accessed on 22 July 2025) and the Fit4Life Program (https://www.unesco.org/en/fit4life, accessed on 22 July 2025), and this is freely available and downloadable.

The PA intervention comprised five components. First, teachers received training on quality physical education and use of the *KaziKidz* toolkit. Second, a Human Movement Science graduate was assigned as a physical education coach to each school to support lesson implementation during the first intervention year; teachers were expected to continue the regimen independently in the second intervention year. However, due to COVID-19-related school closures from March to July 2020, the second year of the intervention could not be implemented as planned. Third, the intervention included one 45 min physical education lesson and one 45 min moving-to-music session per week, plus daily in-class activity breaks. Fourth, schools were provided with basic sports equipment (e.g., skipping ropes, balls, cones, and bean bags) and playground painted games to promote active play. Fifth, teachers received *KaziKidz* materials, including 32 lessons for both physical education and moving-to-music per grade.

Children in the MMNS arm received a daily chewable tablet based on the MixMe™ powder sprinkle provided by DSM Nutritional Products South Africa (Johannesburg, South Africa), administered by class teachers on school days. Details of the composition of the supplement are provided as a Supplemental File (Supplemental Table S1). Children in the PA + placebo and control groups received placebo tablets identical to the MMNS in taste, color, and packaging, ensuring the blinding of participants, teachers, and researchers. All children diagnosed with helminth infections were dewormed with a single 500 mg oral dose of mebendazole prior to the intervention, in line with WHO guidelines [34].

The intervention was intended to span two academic years. Following baseline (T1) assessments (February–April 2019), the first intervention lasted 12 weeks (2 × 45 min lessons per week), interrupted only by school holidays. Post-intervention (T2) data collection occurred from September to November 2019. The second intervention commenced in February 2020 but was halted in March due to COVID-19 lockdowns, which resulted in nationwide school closures on 18 March 2020. Although schools reopened in August 2020, restrictions prevented the continuation of physical education lessons; however, the MMNS intervention resumed for 4 weeks (November–December 2020), with tablets administered 2–3 times per week. The final data collection (T3), initially planned for September–November 2020, was delayed to August 2021. The MMNS intervention continued from February–September 2021 (22 weeks), interrupted only by school holidays. Thus, the first intervention year was implemented as planned, while the second year included only the MMNS arm, extended over a longer period.

### 2.4. Measures

A detailed description of the study methodology has been published in the study protocol [32]. A total of 1151 children were assessed at baseline, 1003 during T2, and 549 at T3. Data were assessed identically at each of the three time-points. At baseline, demographic information (age and sex) was obtained from the school register for all participants. Anthropometric and blood pressure measurements were performed by trained personnel, and nurses collected blood samples using capillary sampling after overnight fasting.

#### 2.4.1. Socioeconomic Status

Socioeconomic status (SES) was assessed via a parental survey with questions related to items about asset ownership (e.g., number of bedrooms and people per household). Details about the items included and the scoring used to estimate SES have been previously described [35]. Principal component analysis was used to generate a wealth index, where items possessed by >95% or <5% of the sample were excluded, with the remaining items used to generate a wealth index score [36]. A high score reflected high SES; quintiles were built based on the wealth index, with quintiles 1 and 5 representing lowest/poorest and highest/wealthiest, respectively.

#### 2.4.2. Anthropometric Measurements

Standing height was measured to the nearest 0.1 cm using a portable Seca stadiometer (Surgical SA; Johannesburg, South Africa), with each participant standing erect with their back against the stadiometer. Body composition and weight were measured by way of bioelectrical impedance analysis (BIA) with overnight fasting using a wireless scale (Tanita MC-580, Tanita Corp; Tokyo, Japan). In light clothing, participants stood on the scale making contact with the metal plates of the machine. Personal data, such as age, sex, and height, were entered into the scale and body weight was measured to the nearest 0.1 kg. Using the impedance and personal data, the scale estimated body fat % and body mass index (BMI), with readings displayed automatically on the Tanita scale. BMI z-scores were computed using Centers for Disease Control and Prevention (CDC)/WHO growth reference data [37].

#### 2.4.3. Device-Based Physical Activity

PA was assessed using an accelerometer device (Actigraph wGT3x-BT; Shalimar, FL, USA) worn around the hip for 7 consecutive days. Children were instructed to continue their daily routine during these 7 days and not take the device off unless they are coming into contact with water. Data were collected in raw 30 Hz acceleration, with a sampling epoch of 15 s counts. Valid wear time was defined as ≥480 min/day accumulated between 6 a.m. and midnight, and children with data for ≥4 weekdays and ≥1 weekend day were included in the analyses. Cut-offs derived from Freedson et al. [38] were used to calculate time spent in MVPA, and data were processed using ActiLife software (version 6.13.4). The present study only included MVPA, expressed in minutes per day (min/day).

#### 2.4.4. Cardiorespiratory Fitness

The 20 m shuttle run test was used to measure CRF. Children ran on a flat surface following a pre-recorded signal, where the initial running speed was set to 8.5 km/h, increasing by 0.5 km/h at each 1 min interval until maximal voluntary exhaustion. Children’s age and the speed at which they stopped running was used to estimate maximum oxygen update (VO_2_max) [39].

#### 2.4.5. CVD Risk Factors

Full lipid profiles (total cholesterol, LDL-C, HDL-C, and triglycerides) and glycated hemoglobin (HbA1c) were analyzed using the point-of-care Alere Afinion AS100 analyser (Abbott Technologies; Abbott Park, IL, USA). Capillary blood sampling was used for the analysis. Blood pressure was measured using an Omron M3 automated blood pressure monitor (Omron Healthcare Europe; Hoofddorp, The Netherlands). Prior to the assessment, children were seated and resting for 5 min. Three measurements were taken with a 1 min interval between each measurement, and the mean of the last two measurements was used for the analyses.

### 2.5. Statistical Analyses

Descriptive characteristics, presented as means (M) and standard deviations (SD), were calculated to describe the sample by intervention group. Models were run for all children before and after adjusting for age, sex, and zBMI. The effect of time and association between exposure and outcome were analyzed using mixed linear models, including the random intercept of school classes to account for the cluster effect. Additionally, the mixed linear model was analyzed adjusting for baseline variables (age, sex, and zBMI), intervention groups, and using the placebo group as reference. Separate analyses were carried out from T1 to T2 and from T1 to T3 to account for the differences between the first- and second-year intervention phases due to the COVID-19 pandemic. We carried out separate analyses without and with intention-to-treat, using the last observation carried forward approach. The results of the intention-to-treat analyses are presented as supplemental files. All analyses were performed using SPSS version 26 for Windows (IBM Corporation; Armonk, NY, USA), and a *p* < 0.05 was considered statistically significant.

## 3. Results

### 3.1. Baseline Characteristics

A total of 1369 children aged 6–12 years from marginalized communities in Gqeberha, South Africa, were enrolled in the study. Of the enrolled participants, 65 dropped out prior to randomization. A total of 1304 children were randomized, based on school class, into one of the four intervention arms and assessed at baseline, with 153 children excluded from the analyses due to invalid PA data (main outcome). Valid data were available for the 1151 children (561 girls, 590 boys) who remained in the study, as depicted in the Consort flow diagram provided as a supplemental file (Supplemental Figure S1).

Participant characteristics are presented in Table 2. At baseline, there were significant differences regarding children’s age, height, and weight (*p* < 0.001) across the intervention groups, with children in the PA + placebo group having the highest mean for all three measures. There were no other significant group differences at baseline.

Table 3 presents descriptive statistics for the baseline outcome variables for the four intervention groups before and after controlling for age, sex, and zBMI. Estimated VO_2_max was significantly different across all four groups (*p* < 0.001), with the placebo group showing the highest mean. Baseline group differences were also observed for BMI and HbA1c, and these differences remained statistically significant even after controlling for age, sex, and zBMI (*p* = 0.001 and 0.017, respectively). Body fat % only revealed significant differences in the adjusted model (*p* = 0.036).

### 3.2. Dropout

Dropout analyses are presented in Supplemental Tables S2 and S3. Of the initial sample, 148 children (13%) dropped out between T1 and T2 and an additional 602 (52%) between T2 and T3. The majority of dropouts during the first intervention year were mainly due to school transfers and incomplete data. The high attrition in the second intervention year was largely attributed to COVID-19-related school closures and the subsequent non-return of many children after schools re-opened in August 2020. Contributing factors included parental concerns about infection risk, confusion due to rotational attendance schedules, and school dropout following the prolonged lockdown. High absenteeism during T3 further hindered follow-up assessments.

There were no significant differences in socio-demographic background at baseline between children who dropped out from T1 to T2 (*p* > 0.05) (Supplemental Table S2). Children who participated at T3 had significantly higher weight than children who dropped out between T1 and T3 (*p* = 0.048) (Supplemental Table S2). Supplemental Table S3 shows in detail the differences in outcome variables for children who did or did not drop out from T1 to T2 and from T1 to T3. Compared to children who dropped out from T1 to T2, children who participated in T2 had significantly lower MVPA and higher HbA1c (*p* = 0.016 and *p* = 0.011, respectively) before and after controlling for age, sex, and zBMI. Compared to children who dropped out from T1 to T3, those who participated at T3 had significantly lower MVPA, and higher BMI, body fat %, and diastolic blood pressure. These results remained significant after controlling for covariates for MVPA (*p* = 0.004) and diastolic blood pressure (*p* = 0.023) but not for BMI and body fat % (*p* > 0.05) (Supplemental Table S3).

### 3.3. First Intervention Year Effects on Outcome Variables (T1–T2)

Table 4 shows that none of the intervention arms had a significant effect on MVPA at T2. The MMNS intervention was associated with a significant increase in estimated VO_2_max and triglycerides after adjusting for baseline age, sex, and zBMI (*p* = 0.001 and *p* = 0.012, respectively). While the PA + placebo intervention was associated with a significant increase in LDL-C (*p* = 0.007). The combined intervention arm, PA + MMNS, significantly decreased HbA1c (*p* < 0.001).

Supplemental Table S4 shows separate analyses carried out with intention-to-treat. Similarly to the analyses without intention-to-treat (Table 4), we found no significant effects of the intervention arms on MVPA. Children in the MMNS group had significantly increased VO_2_max (*p* < 0.001), while those in the PA + placebo group had significantly decreased HDL-C (*p* = 0.043) and triglycerides (*p* = 0.042). The PA + MMNS intervention was associated with a significant decrease in HbA1c (*p* < 0.001).

### 3.4. Second Intervention Year Effects on Outcome Variables (T2–T3)

We found no significant effects of any of the intervention arms on MVPA and VO_2_max at T3. When predicting T3 scores, the PA + MMNS arm was associated with increased body fat % (*p* = 0.003) and decreased HDL-C (*p* = 0.003) (Table 5). While systolic and diastolic blood pressure decreased significantly among children receiving the MMNS (*p* < 0.001), there were no significant effects found for children in the PA + placebo intervention arm for any of the outcome variables compared to the placebo group (as reference).

Separate analyses were carried out with intention-to-treat from T1 to T3 and data are presented as a supplemental file (Supplemental Table S5). Again, there were no significant effects found for MVPA with children assigned to any of the intervention arms. The PA + placebo intervention arm was associated with decreased VO_2_max (*p* = 0.035). Compared to children assigned to the placebo group, peers in the PA + MMNS arm had increased body fat % (*p* = 0.004) at T3, while HDL-C significantly decreased in all intervention arms, PA + MMNS (*p* = 0.010), PA + placebo (*p* = 0.002), and MMNS intervention arms (*p* = 0.032).

## 4. Discussion

This study assessed the effectiveness of a combined school-based PA and MMNS intervention on South African schoolchildren’s MVPA, CRF, and CVD risk factors after one academic school year (T2) and after two academic years (T3). Findings of the study revealed that none of the intervention arms had a significant impact on MVPA levels. Yet, the PA + MMNS arm was effective in reducing HbA1c, while the MMNS arm significantly increased VO_2_max and reduced systolic and diastolic blood pressure. We also found that the PA + MMNS arm was associated with increased body fat % and decreased HDL-C, while the PA + placebo was associated with increased LDL-C and the MMNS arm resulted in increased triglycerides.

After the first-intervention period, we found no significant effect of any of the intervention arms on daily MVPA. Similar findings were reported in a 3-year intervention study among 6–7-year-old children from Copenhagen, Denmark [21]. The study involved doubling physical education lessons (from 90 to 180 min/week), presented twice a week; training physical education teachers; upgrading physical education and play facilities; and providing health education lessons on the importance of PA and healthy eating [21]. The authors attributed the non-significant effect on MVPA to the fact that children’s MVPA was already high at baseline in both the intervention (109–123 min/day) and control group (111–125 min/day) [21]. In the current study sample, children’s mean MVPA at baseline was 81 min/day (95 min/day and 69 min/day for boys and girls, respectively) [40] which is comparable to 6–8-year-olds (80 min/day) [14] but higher compared to 14–18-year-olds in South Africa (51 min/day) [41]. However, in a recent 9-month school-based intervention (120 min of PA per week) involving adolescents from Norway, time spent in MVPA decreased in both the intervention and control groups, but the reduction was significantly smaller in the intervention group (2 vs. 6 min/day) [19]. A decline in MVPA levels during adolescence is not surprising, as PA levels generally decrease from childhood to adolescence [42]. Although some interventions have reported minimal impact on MVPA and only produced small improvements in CRF [43], these findings highlight that these school-based approaches are a start, but they are possibly not enough to have a direct effect outside children’s school life.

We also found that the MMNS intervention was effective in increasing VO_2_max from T1–T2, highlighting that proper nutrition is important for reaching targeted health goals as micronutrient status is known to influence health and body composition [44,45]. These findings align with a 4-month multi-micronutrient intervention study in 7–10-year-old children from India, which found a 17% increase in VO_2_max in the intervention group receiving a fortified choco-malt beverage powder compared to a control group receiving an energy-equivalent unfortified choco-malt beverage powder, and another group receiving no intervention [29]. This is an important finding as CRF is known to track from childhood to adolescence and adulthood [46,47], and evidence has shown that compared to PA, CRF relates more strongly to CVD risk factors in children and adolescents [48].

Regarding CVD risk factors at T2, the MMNS intervention resulted in increased triglyceride levels from the low levels observed at baseline, suggesting that the MMNS may have negatively influenced lipid metabolism, thus increasing the risk of CVD. Previous studies investigating the effects of a school-based PA intervention found that MVPA predicts lower triglycerides in 10-year-old children after a 7-month intervention [13] and after 2 years [22]. However, in our study, the PA intervention on its own did not impact lipid levels; instead, we found increased triglyceride levels in children taking the MMNS. Other studies reported that a MMNS intervention has no impact on triglycerides [30]. The PA + MMNS intervention resulted in a significant decrease in HbA1c, suggesting that a combination of PA and supplementation influenced the risk of type 2 diabetes, while the PA + placebo arm resulted in increased LDL-C. A study investigating the impact of a MMNS on lipidemia in 9–13-year-olds from Brazil found that the supplement significantly reduced total cholesterol, LDL, and glucose [30]. The supplement, consumed as a bar (Nestrovit™), was taken 5 days per week and lasted for a 6-week period and contained vitamins A, E, C, B1, B2, B6, B12, D3, and B5, folate, niacin, biotin, calcium, phosphorus, iron, magnesium, and zinc [30]. The reduction in HbA1c levels in our study in the combined PA + MMNS intervention arm is supported by other studies that reported an association between PA and HbA1c [49] and that a MMNS intervention has potential to reduce HbA1c in children [30], therefore reducing the risk of type 2 diabetes. Although we found our intervention had a negative effect on triglycerides and LDL-C, MMNS have been found to have both short- and long-term benefits on lipid metabolism, thus reducing the risk of CVD in children [30].

After the second intervention year, which was interrupted by the COVID-19 pandemic, we found no significant effects on MVPA at T3 across the different intervention arms. A policy brief on PA and health in children and adolescents in Africa reported a significant decline in activity levels and increase in sedentary behavior due to the COVID-19 pandemic [16]. The closure of schools further denied many children the opportunities to be active, especially in communities where options for PA, sport, and recreation are inaccessible outside of schools [17]. Additionally, the Global Matrix 4.0 Surveillance Study found, among other environmental changes, that COVID-19 restrictions, such as school closures, were associated with an acute decline in opportunities for PA in school-age children [1]. The reduction in PA levels in school-age children has in turn led to biological changes, including reduced physical fitness and increased body fat [1].

Similarly to MVPA, after the interrupted second-year intervention we did not find any significant group differences for VO_2_max between the different intervention arms at T3. Improvements in CRF have been attributed to high intensity PA [7], but there were previous studies that were also unable to report significant improvements in children’s CRF [21,26,50]. However, other interventions have reported significant effects on CRF after 9-month [19], 1-year [18], and 2-year interventions [51]. The studies that did not report a significant impact on CRF attributed their results to insufficient minutes and days per week (180 min, two times a week) allocated to physical education [21] and the low intensity of the intervention [50]. The intervention in the study by Kolle et al. [19] consisted of an additional 120 min/week of PA on top of the compulsory 120 min/week physical education lessons, while Kriemler and colleagues found in their study on Swiss children that increasing physical education volume and intensity (225 min/week) improved CRF and overall PA [18]. Similar results were reported in a 2-year intervention study in Norway implementing MVPA for 300 min/week [51]. Even though the three latter studies were successful in increasing VO_2_max, it should be noted that the participants in these studies came from high-income countries and that data were collected before the COVID-19 pandemic and can therefore not be directly comparable to the children in the current study. Nevertheless, we are aware that school-based interventions are needed now more than before due to declining PA levels, taking into consideration that to improve children’s fitness levels, the frequency, intensity, and duration of the activity should be considered. In our study, the two 45 min lessons allocated for physical education and moving-to-music lessons included the time needed by children to change into gym clothes and organizing the class, which usually consists of 35–50 learners. Although the intervention was designed to engage children in two 45 min lessons of PA a week, time lost in class organization could have shortened this time, meaning the intervention dose was probably lower than the allocated time. The challenge of large class sizes in schools from historically marginalized communities in South Africa has been reported as a barrier to implementing quality physical education [12]. A review of school-based PA interventions suggested that longer duration interventions are needed to effect change in the rate and duration of PA and VO_2_max among children and adolescents aged 6–18 years [52].

Findings of the second-year intervention regarding CVD risk factors showed that the PA + MMNS intervention significantly increased body fat % and decreased HDL-C. It is important to note that only the MMNS was implemented during this intervention period due to the COVID-19 regulations in place. The negative impact of the intervention on body fat % and HDL-C is concerning as these risk factors are relevant in determining cardiovascular health in children and adolescents [47]. Previous studies have reported a reduction in fat mass after a PA intervention [53] and lower BMI and fat mass after a multivitamin and mineral supplement in obese adults [45], suggesting that a combination of PA + MMNS has the potential to reduce body mass and BMI when implemented in an optimal manner. Children participating in the MMNS intervention arm significantly decreased systolic and diastolic blood pressure. This decrease was observed at the 3-year follow-up, which is supported by previous studies where improvements in blood pressure following a PA intervention were reported [21,22]. To our knowledge, the effects of a MMNS intervention on blood pressure in children have not been previously reported. However, MMNS interventions during pregnancy have been widely implemented to reduce low birth weights and improve child growth and health outcomes [54]. A systematic review and meta-analysis examining follow-up data from studies in which pregnant women received MMNS (containing three or more micronutrients) compared to iron and folic acid alone found no evidence of lower blood pressure in their children at follow-up [55]. These findings highlight the need for further research on the potential impact of MMNS interventions, particularly when initiated during childhood, on blood pressure, given its importance as a cardiovascular risk marker.

## 5. Strengths and Limitations

The limitations of the study were the drop-out rate from T1 to T3, which is consistent with previous school-based intervention studies [19,21], although exacerbated by the COVID-19 pandemic. We found that the children who dropped out of the study had higher MVPA and lower BMI, body fat %, and diastolic blood pressure compared to children who participated in T3. We did not assess maturation in our participants aged 6–12 years, which we note as a limitation given that maturation influences CRF levels, especially among girls. Moreover, the COVID-19 pandemic and the closure of schools prevented the implementation of the full second-year intervention, especially in relation to the implementation of the PA component. We were, however, able to continue providing the MMNS from November to December 2020 and again from February to September 2021, but we could not ensure the implementation of physical education at the schools. We are also aware that micronutrient fortification is difficult for school administrators to implement sustainably after the project ends. This makes it even more important that our findings are incorporated into the fortification of normal and regular school meals by school food preparers.

Apart from the limitations identified, several strengths of this study can be noted. First, to our knowledge, this is one of the few studies to investigate the combined effects of PA and MMNS interventions among primary schoolchildren in marginalized communities in Gqeberha, South Africa. Previous studies focusing on MMNS have largely been conducted in high-income settings, making this study particularly novel in its context. Second, we used an objective measure of PA, which reduces bias and enhances the validity of the findings. Third, the study employed a cluster-randomized trial design, strengthening the reliability of the results and their potential applicability to school-based interventions. Finally, our findings contribute new evidence on the relationship between PA, CRF, cardiovascular health, and MMNS in resource-constrained settings, warranting further investigation and long-term follow-up studies.

## 6. Conclusions

Our results indicate that a 90 min/week PA intervention involving teacher training, physical education lessons, and equipment provision may be insufficient to improve daily MVPA, especially when children already achieve MVPA of more than 60 min/day. Increasing intervention frequency, intensity, and duration is needed to improve children’s PA levels, CRF, and cardiovascular health, as shown in previous studies. While we saw some significant effects (that point toward the potential benefits of a PA and/or MMNS intervention), the pattern was not clear and not always as expected. Implementing school-based interventions in marginalized communities remains challenging, and the COVID-19 pandemic further showed the limitations of using schools as intervention settings in times of a pandemic. The pandemic also highlighted the inequalities in access to PA, sport, and recreation needed to achieve PA recommendations. Alternative channels for health promotion are therefore necessary, especially in marginalized communities. Future studies should explore efficient ways for PA and/or MMNS interventions, given the promising results on the impact of the MMNS on CRF, blood sugar, and blood pressure.

## Figures and Tables

**Table 1:** Allocation of classes to intervention arms.

	School 1	School 2	School 3	School 4
Grade 1	PA + placebo	Placebo	PA + MMNS	MMNS
Grade 2	MMNS	PA + placebo	Placebo	PA + MMNS
Grade 3	PA + MMNS	MMNS	PA + placebo	Placebo
Grade 4	Placebo	PA + MMNS	MMNS	PA + placebo

PA = physical activity; MMNS = multi-micronutrient supplementation.

**Table 2:** Sample characteristics for the total sample (sociodemographic background) and differences between intervention groups at baseline.

Intervention Group							
	Overall (n = 1151)	PA + MMNS (n = 258)	PA + Placebo (n = 307)	MMNS (n = 283)	Placebo (n = 303)	F	*p*-Value
Child Characteristics	M (SD)	M (SD)	M (SD)	M (SD)	M (SD)		
Age (years)	8.3 (1.4)	8.4 (1.3)	8.8 (1.5)	8.1 (1.5)	7.9 (1.3)	**23.47**	<0.001 **
Sex, girls, n (%)	561 (49)	130 (23)	159 (28)	129 (23)	143 (26)	2.84	0.416
SES	0.75 (0.16)	0.73 (0.15)	0.77 (0.16)	0.76 (0.15)	0.75 (0.16)	1.61	0.187
Height (cm)	124.7 (9.3)	125.2 (9.1)	127.5 (9.9)	123.5 (8.2)	122.5 (9.0)	**17.40**	<0.001 **
Weight (kg)	25.4 (6.8)	25.8 (6.8)	27.4 (8.4)	24.6 (5.4)	23.7 (5.5)	**16.82**	<0.001 **

PA = Physical activity; MMNS = Multi-micronutrient supplementation; SES = Socioeconomic status. ** *p* < 0.001.

**Table 3:** Descriptive statistics for outcome variables in the total sample and differences between intervention groups.

Intervention and Control Groups									
	Overall (n = 1151)	PA + MMNS (n = 258)	PA + Placebo (n = 307)	MMNS (n = 283)	Placebo (n = 303)	Unadjusted Model		Adjusted Model ^a^	
Outcome Variables	M (SD)	M (SD)	M (SD)	M (SD)	M (SD)	F	*p*-Value	F	*p*-Value
MVPA (min/day)	82.37 (28.00)	82.48 (27.90)	79.60 (29.24)	83.69 (28.73)	83.85 (25.98)	1.50	0.214	0.53	0.659
Estimated VO_2_max (mL/kg/min)	47.52 (3.85)	47.21 (3.65)	46.89 (4.18)	47.78 (3.70)	48.16 (3.70)	**6.44**	<0.001 **	0.75	0.522
BMI (kg/m^2^)	16.08 (2.62)	16.22 (2.61)	16.47 (3.26)	16.03 (2.27)	16.63 (2.12)	**5.44**	<0.001 **	**5.76**	<0.001 **
zBMI	−0.12 (1.25)	−0.06 (1.29)	−0.11 (1.36)	−0.04 (1.18)	−0.25 (1.17)	1.63	0.182	1.89	0.129
Body fat (%)	22.61 (5.30)	22.45 (5.13)	23.14 (6.23)	22.71 (4.87)	22.12 (4.75)	1.92	0.125	**2.85**	0.036 *
Total cholesterol (mmol/L)	3.63 (0.64)	3.59 (0.61)	3.68 (0.64)	3.70 (0.64)	3.58 (0.65)	2.05	0.106	1.16	0.325
LDL-C (mmol/L)	2.07 (0.53)	2.01 (0.52)	2.11 (0.54)	2.10 (0.51)	2.05 (0.54)	1.71	0.164	1.02	0.385
HDL-C (mmol/L)	1.23 (0.31)	1.23 (0.29)	1.23 (0.32)	1.26 (0.31)	1.21 (0.32)	1.10	0.349	1.05	0.369
Triglycerides (mmol/L)	0.76 (0.31)	0.79 (0.31)	0.77 (0.25)	0.74 (0.27)	0.74 (0.39)	1.55	0.199	0.72	0.540
HbA1c (%)	5.42 (0.26)	5.43 (0.24)	5.44 (0.31)	5.37 (0.23)	5.44 (0.24)	**3.43**	0.017 *	**3.43**	0.017 *
Systolic blood pressure (mmHg)	102.0 (11.9)	102.6 (11.5)	101.9 (11.8)	102.6 (12.3)	101.0 (11.8)	1.11	0.345	0.91	0.438
Diastolic blood pressure (mmHg)	63.9 (9.4)	63.9 (8.4)	63.8 (8.9)	64.1 (9.5)	63.6 (10.6)	0.14	0.937	0.38	0.768

PA = Physical activity; MMNS = Multi-micronutrient supplementation; MVPA = Moderate-to-vigorous physical activity; VO_2_max = Maximal oxygen uptake; BMI = Body mass index; zBMI = BMI-for-age z-score; LDL-C = Low-density lipoprotein cholesterol; HDL-C = High-density lipoprotein cholesterol; HbA1c = Glycated hemoglobin. ^a^ Models adjusted for age, sex, and zBMI. * *p* < 0.05, ** *p* < 0.001.

**Table 4:** Mixed linear model to predict T2 outcomes for each intervention arm in comparison with placebo.

Intervention Groups ^a^						
	PA + MMNS		PA + Placebo		MMNS	
Outcome Variables	Coefficient	95% CI	Coefficient	95% CI	Coefficient	95% CI
MVPA (min/day)	2.31	(−1.26; 5.87)	−1.28	(−4.82; 2.26)	−1.42	(−4.94; 2.10)
Estimated VO_2_max (mL/kg/min)	−0.00	(−0.54; 0.53)	−0.19	(−0.72; 0.34)	**1.21**	(0.68; 1.73) **
Body fat (%)	0.35	(−0.09; 0.80)	−0.31	(−0.76; 0.14)	−0.04	(−0.48; 0.41)
Total cholesterol (mmol/L)	−0.01	(−0.10; 0.09)	0.04	(−0.07; 0.14)	0.02	(−0.08; 0.12)
LDL-C (mmol/L)	0.05	(−0.03; 0.13)	**0.11**	(0.03; 0.20) *	0.05	(−0.03; 0.13)
HDL-C (mmol/L)	−0.02	(−0.08; 0.03)	−0.04	(−0.10; 0.01)	−0.04	(−0.10; 0.01)
Triglycerides (mmol/L)	0.03	(−0.03; 0.08)	−0.03	(−0.09; 0.03)	**0.07**	(0.02; 0.13) *
HbA1c (%)	**−0.07**	(−0.11; −0.03) **	−0.04	(−0.08; 0.00)	−0.00	(−0.04; 0.04)
Systolic blood pressure (mmHg)	−0.47	(−2.57; 1.64)	0.53	(−1.56; 2.61)	−1.87	(−3.94; 0.21)
Diastolic blood pressure (mmHg)	0.91	(−0.85; 2.67)	1.64	(−0.11; 3.39)	−1.36	(−3.10; 0.37)

PA = Physical activity; MMNS = Multi-micronutrient supplementation; MVPA = Moderate-to-vigorous physical activity; VO_2_max = Maximal oxygen uptake; LDL-C = Low-density lipoprotein cholesterol; HDL-C = High-density lipoprotein cholesterol; HbA1c = Glycated hemoglobin. ^a^ The placebo group is used as a reference, controlling for baseline age, sex, and zBMI. Class considered as random intercept. * *p* < 0.05, ** *p* < 0.001.

**Table 5:** Mixed linear model to predict T3 outcomes for each intervention arm in comparison with placebo.

Intervention Groups ^a^						
	PA + MMNS		PA + Placebo		MMNS	
Outcome Variables	Coefficient	95% CI	Coefficient	95% CI	Coefficient	95% CI
MVPA (min/day)	−1.99	(−7.31; 3.34)	1.33	(−4.04; 6.69)	3.51	(−2.01; 9.03)
Estimated VO_2_max (mL/kg/min)	0.04	(−0.68; 0.75)	−0.17	(−0.88; 0.54)	0.19	(−0.55; 0.92)
Body fat (%)	**1.58**	(0.55; 2.61) *	−0.06	(−1.11; 1.00)	0.48	(−0.58; 1.53)
Total cholesterol (mmol/L)	−0.12	(−0.26; 0.03)	−0.12	(−0.26; 0.03)	−0.04	(−0.18; 0.11)
LDL-C (mmol/L)	0.06	(−0.05; 0.17)	−0.04	(−0.15; 0.07)	0.02	(−0.09; 0.14)
HDL-C (mmol/L)	**−0.12**	(−0.19; −0.04) *	−0.07	(−0.15; 0.01)	−0.08	(−0.16; 0.00)
Triglycerides (mmol/L)	0.01	(−0.15; 0.16)	0.05	(−0.11; 0.21)	0.06	(−0.10; 0.22)
HbA1c (%)	0.01	(−0.04; 0.06)	−0.00	(−0.05; 0.05)	0.03	(−0.02; 0.08)
Systolic blood pressure (mmHg)	10.03	(−7.52; 27.58)	−13.01	(−30.40; 4.39)	**−36.53**	(−53.83; −19.23) **
Diastolic blood pressure (mmHg)	7.97	(−6.02; 21.95)	−10.47	(−24.34; 3.40)	**−29.18**	(−42.96; −15.40) **

PA = Physical activity, MMNS = Multi-micronutrient supplementation, MVPA = Moderate-to-vigorous physical activity, VO_2_max = Maximal oxygen uptake, LDL-C = Low-density lipoprotein cholesterol, HDL-C = High-density lipoprotein cholesterol, HbA1c = Glycated hemoglobin. ^a^ The placebo group is used as a reference, controlling for baseline age, sex, zBMI. Class considered as random intercept. * *p* < 0.05, ** *p* < 0.001.

## Data Availability

All data relevant to the study are included in the article or as Supplementary Information. The datasets used and/or analyzed during the current study are available from the corresponding author upon reasonable request.

## Supplementary Materials

The following supporting information can be downloaded at: https://www.mdpi.com/article/10.3390/children12101352/s1, Supplemental Figure S1. Consort flow chart; Supplemental Table S1. Composition of the multi-micronutrient supplement; Supplemental Table S2. Differences in socio-demographic background between schoolchildren who did/did not drop out from (a) T1 to T2 and (b) T1 to T3; Supplemental Table S3. Differences in outcome variables between children who did/did not drop out from (a) T1 to T2 and (b) T1 to T3; Supplemental Table S4. Mixed linear model predicting T2 scores, after controlling for T1, age, sex, zBMI, and intervention group, with intention-to-treat; Supplemental Table S5. Mixed linear model predicting T3 scores, after controlling for T1, age, sex, zBMI, and intervention group, with intention-to-treat.

## Abbreviations

The following abbreviations are used in this manuscript:

BMI	Body mass index
zBMI	Body mass index z-score
CAPS	Curriculum and assessment policy statement
COVID-19	Coronavirus disease 2019
CVD	Cardiovascular disease
Fit4Life	Fit for Life Program of UNESCO
HAKSA	Healthy active kids South Africa
HbA1c	Glycated hemoglobin
HDL	High-density lipoprotein cholesterol
LDL-C	Low-density lipoprotein cholesterol
LMICs	Low- and middle-income countries
M	Means
MVPA	Moderate-to-vigorous intensity physical activity
SD	Standard deviation
SES	Socioeconomic status
T1	Baseline measurement
T2	Follow-up measurement
T3	End-line measurement
QPE	Quality Physical Education Progamme of UNESCO
VO_2_max	Maximal oxygen uptake
WHO	World Health Organization
