# Supplementary material for: A Combined Physical Activity and Multi-Micronutrient Supplementation Intervention in South African Primary Schools: Effects on Physical Activity, Fitness, and Cardiovascular Disease Risk Factors
Source: Children (Basel). 2025 Oct 9;12(10):1352. doi: 10.3390/children12101352 (PMC12562825; doi:10.3390/children12101352)
Supplement: Supplementary file 1 [file children-12-01352-s001.zip › Supplemental Figure S1.pdf]

CONSORT
TRANSPARENT REPORTING of TRIALS

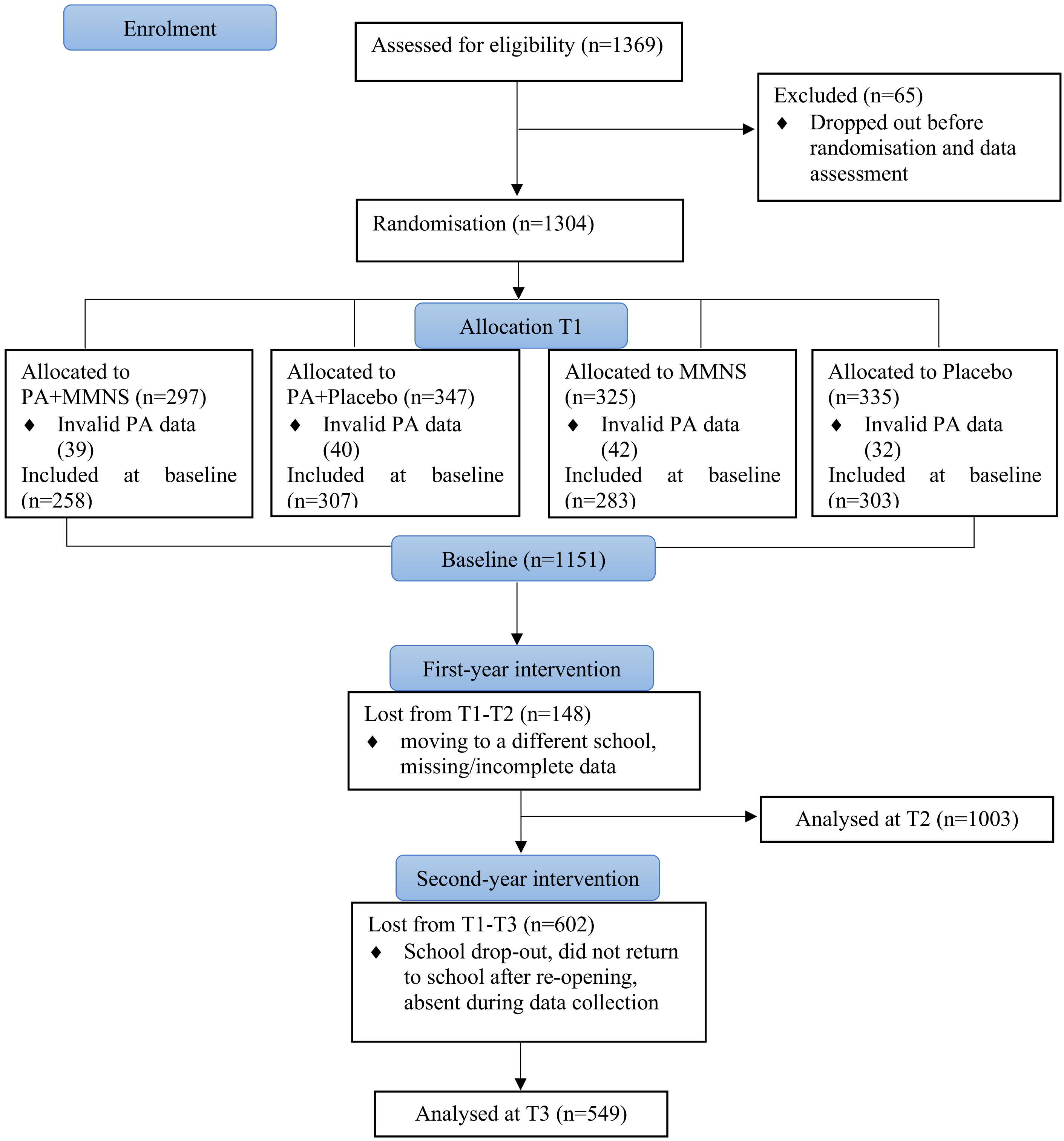

Figure S1. CONSORT flow diagram for schoolchildren included in the analysis

PA=Physical activity, MMNS=Multi-micronutrient supplementation, T1=baseline, T2=post-intervention, T3=follow-up
