# Supplementary material for: A Combined Physical Activity and Multi-Micronutrient Supplementation Intervention in South African Primary Schools: Effects on Physical Activity, Fitness, and Cardiovascular Disease Risk Factors
Source: Children (Basel). 2025 Oct 9;12(10):1352. doi: 10.3390/children12101352 (PMC12562825; doi:10.3390/children12101352)
Supplement: Supplementary file 1 [file children-12-01352-s001.zip › Supplemental Table S1.pdf]

**Supplemental Table S1.** Composition of the multi-micronutrient supplement

| No. | Nutrient | Average per 1 tablet |
|---|---|---|
| 1 | β-carotene (as BetTab 20%S) | 3.6 mg |
| 2 | Vitamin D | 400 IU/10 mcg |
| 3 | Vitamin E | 9 mg TE |
| 4 | Vitamin K | 30 mcg |
| 5 | Vitamin C | 60 mg |
| 6 | Vitamin B1 Thiamine | 1.1 mg |
| 7 | Vitamin B2 Riboflavin | 1.3 mg |
| 8 | Vitamin B6 Pyriodoxine | 0.5 mg |
| 9 | Vitamin B12 | 1.2 mcg |
| 10 | Folic acid | 200 mcg |
| 11 | Niacinamide | 8 mg |
| 12 | Iron (added as Fe-EDTA) | 8 mg |
| 13 | Zinc (added as zinc oxide) | 5 mg |
| 14 | Selenium (added as sodium selenite anhydrous) | 20 mcg |
| 15 | Iodine (added as potassium iodate) | 100 mcg |

Ingredients 1–15 are nutrients and were produced with an overage to ensure required amounts during the shelf-life.

Inactive ingredients: sugar, citric acid, sorbitol, non-nutritive sweetener, flavour.

Placebo tablets do not contain nutrients, but have colorants added.

Dosage and directions: one tablet daily during the first school lesson. The tablets were stored in a climate-controlled storage room at the respective research institution and were provided to school on a fortnightly basis
