# Supplementary material for: A Combined Physical Activity and Multi-Micronutrient Supplementation Intervention in South African Primary Schools: Effects on Physical Activity, Fitness, and Cardiovascular Disease Risk Factors
Source: Children (Basel). 2025 Oct 9;12(10):1352. doi: 10.3390/children12101352 (PMC12562825; doi:10.3390/children12101352)
Supplement: Supplementary file 1 [file children-12-01352-s001.zip › Supplemental Table S2.pdf]

Supplemental Table S2. Differences in socio-demographic background between students who did/did not drop out from (a) T1 to T2 and (b) T1 to T3

	**(a) T1 to T2**			
	**Dropout (*n*=148)**	**Not dropout (*n*=1003)**		
**Participants characteristics**	***M* (*SD*)**	***M* (*SD*)**	***F***	***p-value***
Age (years)	8.39 (1.55)	8.09 (4.99)	0.56	0.454
Sex, girls, *n* (%)	71 (13)	490 (87)	0.31	0.579
SES	0.76 (0.15)	0.75 (0.16)	0.15	0.699
Height (cm)	124.47 (8.97)	124.76 (9.31)	0.12	0.732
Weight (kg)	25.32 (6.67)	25.40 (6.80)	0.02	0.901
	**(b) T1 to T3**			
	**Dropout (*n*=602)**	**Not dropout (*n*=549)**		
Age (years)	7.93 (6.32)	8.34 (1.39)	2.15	0.143
Sex, girls, *n* (%)	280 (50)	281 (50)	3.09	0.079
SES	0.75 (0.16)	0.75 (0.15)	0.07	0.789
Height (cm)	124.46 (9.28)	124.72 (9.26)	0.95	0.329
Weight (kg)	25.00 (6.41)	25.80 (7.15)	**3.92**	**0.048 ***

SES=Socio-economic status, T1=baseline, T2=post-intervention, T3=follow-up *p<0.05
