# Supplementary material for: A Combined Physical Activity and Multi-Micronutrient Supplementation Intervention in South African Primary Schools: Effects on Physical Activity, Fitness, and Cardiovascular Disease Risk Factors
Source: Children (Basel). 2025 Oct 9;12(10):1352. doi: 10.3390/children12101352 (PMC12562825; doi:10.3390/children12101352)
Supplement: Supplementary file 1 [file children-12-01352-s001.zip › Supplemental Table S3.pdf]

Supplemental Table S3. Differences in outcome variables between children who did/did not drop out from (a) T1 to T2 and (b) T1 to T3

	Dropout (*n*=148)	Not dropout (*n*=1003)	Unadjusted model		Adjusted model[a]	
**(a) T1 to T2**						
**Participants characteristics**	***M* (*SD*)**	***M* (*SD*)**	***F***	***p-value***	***F***	***p-value***
MVPA (min/day)	87.94 (30.23)	81.45 (27.54)	**7.22**	**0.007** *	**5.78**	**0.016** *
Estimated VO2max (ml/kg/min)	47.16 (4.05)	47.57 (3.82)	1.47	0.225	0.40	0.529
BMI (kg/m^2)	16.16 (2.73)	16.08 (2.61)	0.13	0.721	0.09	0.768
*z*BMI	-0.09 (1.28)	-0.13 (1.25)	0.11	0.746	0.12	0.728
Body fat (%)	22.77 (5.72)	22.60 (5.26)	0.12	0.726	0.61	0.437
Total cholesterol (mmol/L)	3.72 (0.65)	3.62 (0.63)	2.13	0.145	2.89	0.089
LDL-C (mmol/L)	2.11 (0.50)	2.06 (0.53)	0.84	0.361	1.48	0.224
HDL-C (mmol/L)	1.26 (0.32)	1.22 (0.31)	1.52	0.219	1.98	0.160
Triglycerides (mmol/L)	0.76 (0.27)	0.76 (0.32)	0.03	0.861	0.23	0.633
HbA1c (%)	5.36 (0.21)	5.43 (0.26)	**6.75**	**0.010** *	**6.53**	**0.011** *
Systolic blood pressure (mmHg)	102.15 (11.50)	101.96 (11.91)	0.03	0.857	0.03	0.859
Diastolic blood pressure (mmHg)	63.43 (8.62)	63.90 (9.54)	0.33	0.563	0.30	0.586
**(b) T1 to T3**						
	**Dropout (*n*=602)**	**Not dropout (*n*=549)**	**Unadjusted model**		**Adjusted model[a]**	
MVPA (min/day)	84.95 (28.36)	79.39 (27.30)	**11.48**	**<0.001****	**8.53**	**0.004***
Estimated VO2max (ml/kg/min)	47.50 (3.90)	47.54 (3.81)	0.04	0.849	0.42	0.515
BMI (kg/m^2)	15.94 (2.49)	16.25 (2.76)	**3.87**	**0.049** *	0.02	0.884
*z*BMI	-0.19 (1.24)	-0.05 (1.26)	3.41	0.065	3.46	0.063
Body fat (%)	22.15 (5.08)	23.14 (5.51)	**9.76**	**0.002** *	2.58	0.108
Total cholesterol (mmol/L)	3.65 (0.65)	3.61 (0.62)	1.25	0.264	2.07	0.150
LDL-C (mmol/L)	2.08 (0.55)	2.06 (0.51)	0.16	0.692	0.54	0.465
HDL-C (mmol/L)	1.24 (0.32)	1.22 (0.30)	0.54	0.461	0.62	0.433
Triglycerides (mmol/L)	0.76 (0.27)	0.76 (0.34)	0.14	0.712	0.63	0.427
HbA1c (%)	5.42 (0.24)	5.43 (0.27)	0.47	0.496	0.40	0.526
Systolic blood pressure (mmHg)	101.40 (11.65)	102.61 (12.05)	2.96	0.085	2.32	0.128
Diastolic blood pressure (mmHg)	63.15 (9.09)	64.59 (9.72)	**6.70**	**0.010** *	**5.15**	**0.023** *

MVPA=Moderate-to-vigorous physical activity, VO_2max=Maximal oxygen uptake, BMI=Body mass index, *z*BMI=BMI-for-age Z-score, LDL-C=Low-density lipoprotein cholesterol, HDL-C=High-density lipoprotein cholesterol, HbA1c=Glycated haemoglobin, T1=baseline, T2=post-intervention, T3=follow-up

[a]Models adjusted for age, sex and *z*BMI

*p<0.05, **p<0.001
