# Supplementary material for: A Combined Physical Activity and Multi-Micronutrient Supplementation Intervention in South African Primary Schools: Effects on Physical Activity, Fitness, and Cardiovascular Disease Risk Factors
Source: Children (Basel). 2025 Oct 9;12(10):1352. doi: 10.3390/children12101352 (PMC12562825; doi:10.3390/children12101352)
Supplement: Supplementary file 1 [file children-12-01352-s001.zip › Supplemental Table S4.pdf]

Supplemental Table S4. Mixed linear model predicting T2 scores, after controlling for T1, age, sex, *z*BMI, and intervention group, with intention-to-treat

	**Intervention group**					
	**PA+MMNS**		**PA+Placebo**		**MMNS**	
**Outcome variables**	***B-coefficient***	***95% CI***	***B-coefficient***	***95% CI***	***B-coefficient***	***95% CI***
MVPA (min/day)	2.87	(-0.54; 6.28)	-1.55	(-4.89; 1.80)	-1.96	(-5.27; 1.35)
Estimated VO_2max (ml/kg/min)	-0.00	(-0.49; 0.49)	-0.20	(-0.68; 0.28)	**1.28**	**(0.80; 1.75)** **
Body fat (%)	0.28	(-0.12; 0.68)	-0.35	(-0.74; 0.05)	-0.03	(-0.42; 0.36)
Total cholesterol (mmol/L)	-0.02	(-0.10; 0.07)	0.01	(-0.07; 0.10)	0.01	(-0.08; 0.09)
LDL-C (mmol/L)	0.03	(-0.04; 0.09)	0.09	(0.02; 0.15)	0.03	(-0.04; 0.)
HDL-C (mmol/L)	-0.02	(-0.07; 0.03)	**-0.05**	**(-0.10; -0.00)** *	-0.04	(-0.09; 0.01)
Triglycerides (mmol/L)	-0.01	(-0.06; 0.04)	**-0.05**	**(-0.11; -0.00)** *	0.04	(-0.01; 0.09)
HbA1c (%)	**-0.07**	**(-0.10; -0.04)** **	-0.03	(-0.06; 0.01)	0.00	(-0.03; 0.04)
Systolic blood pressure (mmHg)	-0.45	(-2.40; 1.51)	0.51	(-1.42; 2.43)	-1.63	(-3.54; 0.28)
Diastolic blood pressure (mmHg)	1.04	(-0.63; 2.72)	1.38	(-0.27; 3.02)	-0.77	(-2.40; 0.86)

PA=Physical activity, MMNS=Multi-micronutrient supplementation, MVPA=Moderate-to-vigorous physical activity, VO_2max=Maximal oxygen uptake, LDL-C=Low-density lipoprotein cholesterol, HDL-C=High-density lipoprotein cholesterol, HbA1c=Glycated haemoglobin

[a]The placebo group is used as reference. Class considered as random intercept

*p<0.05, **p<0.001
