# Supplementary material for: A Combined Physical Activity and Multi-Micronutrient Supplementation Intervention in South African Primary Schools: Effects on Physical Activity, Fitness, and Cardiovascular Disease Risk Factors
Source: Children (Basel). 2025 Oct 9;12(10):1352. doi: 10.3390/children12101352 (PMC12562825; doi:10.3390/children12101352)
Supplement: Supplementary file 1 [file children-12-01352-s001.zip › Supplemental Table S5.pdf]

Supplemental Table S5. Mixed linear model predicting T3 scores, after controlling for T1, age, sex, *z*BMI, and intervention group, with intention-to-treat

	Intervention group					
	PA+MMNS		PA+Placebo		MMNS	
**Outcome variables**	***B-coefficient***	***95% CI***	***B-coefficient***	***95% CI***	***B-coefficient***	***95% CI***
MVPA (min/day)	-3.13	(-7.03; 0.77)	-1.99	(-5.83; 1.83)	-0.18	(-3.97; 3.62)
Estimated VO_2max (ml/kg/min)	-0.25	(-0.86; 0.35)	**-0.64**	**(-1.24; -0.05)** *	0.49	(-0.10; 1.08)
Body fat (%)	**0.80**	**(0.25; 1.35)** *	-0.10	(-0.64; 0.44)	0.17	(-0.37; 0.70)
Total cholesterol (mmol/L)	-0.09	(-0.18; 0.01)	-0.08	(-0.18; 0.01)	-0.03	(-0.12; 0.07)
LDL-C (mmol/L)	0.00	(-0.07; 0.07)	0.02	(-0.06; 0.09)	0.02	(-0.05; 0.09)
HDL-C (mmol/L)	**-0.07**	**(-0.12; -0.02)** *	**-0.08**	**(-0.13; -0.03)** *	**-0.06**	**(-0.11; -0.01)** *
Triglycerides (mmol/L)	-0.00	(-0.09; 0.08)	-0.01	(-0.10; 0.07)	0.01	(-0.07; 0.09)
HbA1c (%)	-0.03	(-0.07; 0.00)	0.00	(-0.04; 0.04)	0.01	(-0.03; 0.04)
Systolic blood pressure (mmHg)	0.45	(-1.49; 2.40)	-0.16	(-2.07; 1.76)	-1.25	(-3.15; 0.65)
Diastolic blood pressure (mmHg)	0.90	(-0.61; 2.41)	0.99	(-0.50; 2.48)	0.53	(-0.95; 2.00)

PA=Physical activity, MMNS=Multi-micronutrient supplementation, MVPA=Moderate-to-vigorous physical activity, VO_2max=Maximal oxygen uptake, LDL-C=Low-density lipoprotein cholesterol, HDL-C=High-density lipoprotein cholesterol, HbA1c=Glycated haemoglobin

[a]The placebo group is used as reference. Class considered as random intercept

*p<0.05, **p<0.001
